# Cellular Structure Optimization in Microwave-Assisted Foaming of Acrylated Epoxidized Soybean Oil

**DOI:** 10.1021/acsomega.5c09441

**Published:** 2025-11-13

**Authors:** Adriano Vignali, Fabio Bertini, Salvatore Iannace

**Affiliations:** Institute of Chemical Sciences and Technologies “Giulio Natta” (SCITEC), National Research Council (CNR), Via A. Corti 12, Milano 20133, Italy

## Abstract

This study demonstrates the effective and rapid foaming of biobased acrylated epoxidized soybean oil (AESO) resins using microwave technology. Utilizing a multimode household microwave oven, AESO could be heated up to 200 °C, enabling the simultaneous decomposition of a chemical blowing agent and cross-linking reaction. Rheometric analysis under nonisothermal conditions revealed that the peroxide-initiated curing of the AESO proceeded rapidly in the 100–120 °C range, with a 6 order of magnitude increase in complex viscosity. Further isothermal rheometric tests at 80 °C successfully demonstrated the ability to precure AESO resin to various degrees of polymerization, both before and beyond its gel point. Foams produced by 2 min of microwave irradiation at 800 W exhibited significantly different morphologies depending on the degree of precuring. The optimal cellular structure was achieved with resin precured just below its gel point, yielding a foam with an apparent density of 0.37 g/cm^3^, an average cell diameter of 190 μm, and an elastic modulus of 1.84 MPa. These findings highlight the potential of microwave processing for the efficient production of biobased AESO foams with tailored properties.

## Graphical abstract

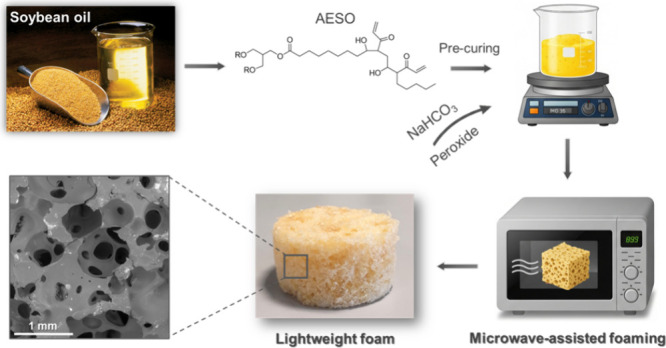

## Introduction

Polymeric foams are lightweight materials characterized by a dispersed gaseous phase within a polymeric matrix.^1,2^ These structures are widely utilized across various industries due to their ability to significantly reduce product weight while offering the flexibility to customize the mechanical and functional properties to meet specific requirements. The properties of these foams are influenced not only by the type of polymer employed but also by the foam’s structural characteristics, including the degree of expansion and foam morphology (e.g., the ratio of open to closed cells, cell dimensions, and cell size distribution). In comparison to their solid counterparts, polymeric foams often exhibit enhanced properties such as superior impact resistance, toughness, fatigue resistance, thermal stability, dielectric strength, and better performance in thermal and acoustic insulation. Consequently, foamed materials find applications in a diverse range of fields, including packaging,^3,4^ automotive,^5^ construction,^6^ insulation,^7^ furniture,^8^ and tissue engineering.^9^

Typically, polymeric foams are produced using gas foaming techniques that employ physical blowing agents (e.g., carbon dioxide, nitrogen, and hydrocarbons) to create bubbles within a softened polymer matrix. The stages involved in gas foaming include (a) solubilization of the blowing agent in the polymer under high pressure to create a polymer/gas solution, (b) bubble nucleation via pressure and/or temperature changes, (c) bubble growth, and (d) solidification of the foam structure.^10^ The specific properties of the polymer/gas system including thermodynamics, solution physics, interface phenomena, and rheology will influence the final porous structures.

In thermosetting polymeric foams, such as, for example, in thermosetting polyurethane foams, the solubilization step of the blowing agent is typically absent since bubble formation occurs during the generation of blowing gases directly within the matrix, rather than the separation of gas from a polymer/gas solution. In those cases where carbon dioxide is used as a physical blowing agent, one should take into account that CO_2_ can influence the chemical reactions occurring during the resin curing process. Several studies have highlighted that CO_2_ often leads to catalyst deactivation, ultimately altering the polymerization reaction kinetics.^11–13^ Another critical factor in developing foaming strategies for thermosetting polymers is the control of bubble nucleation and growth during the viscosity increase due to the curing of the resin. This can be achieved by tuning the processing conditions, such as pressure and/or temperature profiles, during the foaming process.^14^

Vegetable oils represent an attractive class of renewable, abundant, and economical starting materials for generating sustainable polymers with diverse structures, properties, and applications. Their intrinsic molecular complexity, arising from their carbon backbones, inherent unsaturations, and various functional groups, offers significant potential for the development of novel materials.^15^ Furthermore, the utilization of waste cooking oil as a precursor for biobased products presents a compelling, cost-effective alternative to virgin vegetable oils, aligning with principles of circular economy and waste valorization.^16^

However, the fatty acid profile significantly differentiates vegetable oils of various origins, impacting both the carbon chain length and the degree of unsaturation along the chains. Specifically, the number of carbon–carbon double bonds critically influences the preparation of cross-linked materials. A high level of unsaturation is often a prerequisite for highly cross-linked systems, such as epoxy or epoxy-derived thermosetting resins.^17^

Soybean oil, recognized globally as a highly abundant renewable vegetable oil, can be readily converted to an epoxy resin through an epoxidation reaction. The resulting epoxidized soybean oil (ESO) has been employed in the production of biobased thermosetting epoxy polymers^18–20^ and epoxy foams.^21–23^ Despite significant advancements in developing thermosetting materials from these biomass-derived feedstocks, a portion of the final product often still originates from petroleum.

The addition of acrylic acid to ESO to yield acrylated epoxidized soybean oil (AESO) enhances the reactivity of soybean oil, introducing additional polymerizable functionalities like acrylate groups that readily cross-link through free-radical polymerization. Moreover, AESO can be polymerized with biobased terpenic comonomers that increase the total amount of biobased feedstock, enabling the development of thermosetting resins with up to 84% of the overall carbon from biomass and with performance comparable to AESO thermosets obtained with comonomers deriving from petrochemical sources.^24^

Very scarce literature is available on AESO foaming.^11,25–27^ Bonnaillie and Wool investigated that the fabrication of AESO foams utilized pressurized CO_2_ as a foaming agent. In this approach, the density of the cured foam was regulated by applying a partial vacuum just prior to gelation. The final foam morphology was critically influenced by the extent of resin curing when the vacuum was applied to promote bubble nucleation and growth.^11^

In the present study, microwave-assisted biobased foams of AESO were obtained by using a nontoxic sodium bicarbonate as a chemical blowing agent. Unlike conventional heating, microwave (MW) energy directly couples with polar molecules, enabling rapid and volumetric heating of the resin.^28^ Therefore, MW irradiation offers a precise and efficient method for temperature control during the cross-linking of foams. This controlled internal heating allows for fine-tuning of the reaction kinetics, ensuring fast and uniform curing throughout the foam structure and potentially mitigating issues like incomplete cross-linking that can arise with external heating methods.

Although the use of microwave processing is an expanding field of study, only a limited number of studies are dedicated specifically to foaming materials^29^ and these are mainly based on starch-based foams,^30–33^ biocomposite foams with multimodal cellular structures based on cork granulates and egg white proteins,^34^ phenolic foams,^35^ expandend polystyrene foams,^36^ and rubbers.^37,38^ No studies have been reported so far on MW-assisted AESO foaming.

The influence of the MW processing parameters on the resulting cellular morphology of the AESO foams was investigated in this work. Specifically, cellular growth was observed to be promoted under conditions where the viscosity of the liquid phase was sufficiently elevated to reduce coalescence and coarsening and where the cross-link density was adequately low to permit foam expansion. Finally, the thermal stability and mechanical properties of the AESO-based foams were studied by thermogravimetric analysis and compression tests.

## Experimental Section

### Materials

Acrylated epoxidized soybean oil, *tert*-butyl peroxybenzoate (Luperox P), and sodium bicarbonate (NaHCO_3_) were purchased by Merck and used as received.

### Process Optimization and Foam Preparation

In the preparation of MW-assisted foams, the desired amount of AESO (9.5 g) was placed in a cylindrical-shaped silicon mold (diameter = 4 cm, height = 2 cm) and mixed with a spatula alongside 0.0125 wt % radical initiator Luperox P and 5 wt % foaming agent NaHCO_3_. To obtain precured resins at different stages relative to their gelation time, the mixture was first subjected to stirring and heating at 80 °C for various intervals on a hot plate. After this precuring, the resin underwent rapid microwave heating at 800 W for 2 min in a Samsung MG23F302TAK household microwave oven (Scheme 1). This oven (2450 MHz frequency, 800 W maximum power) features a triple distribution system, utilizing three antennas to achieve an optimized microwave field distribution within the cavity. To optimize the MW-assisted foaming process, a preliminary investigation into MW heating efficiency was undertaken. This involved acquiring thermal images at several time steps using a Seek CompactPro thermal imaging camera, equipped with a 320 × 240 thermal sensor and capable of detecting temperatures from −40 to 300 °C. Microwave heating experiments were also performed in a MW system CEM Discover 2.0 at a single power of 50 W for 90 s for AESO, water, and soybean oil (SO).

**Scheme 1:**
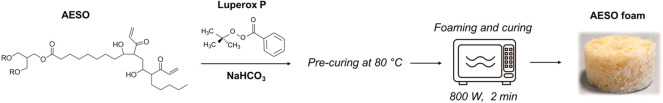
Schematic Representation of the AESO Foam Preparation

### Characterization

The curing kinetics of AESO was studied by a TA Instruments AR 2000 rotational rheometer both with temperature sweep at a heating rate of 5 °C/min and time sweep at three different temperatures (60, 70, and 80 °C) at a single frequency and strain of 1 rad/s and 1%, respectively. The rheometer was equipped with parallel plates with a diameter of 25 mm. The apparent density of foams was determined by measuring the dimensions of samples with a digital caliper (accuracy = 0.01 mm) and weighing the samples with a precision balance (accuracy = 0.01 mg). The morphology of foams was investigated by a Thermo Fisher Scientific Phenom XL G2 scanning electron microscope operating at 10 kV. Before the observations, the foams were blade-cut from the middle. The Phenom Image Viewer software was employed to measure the pore sizes. For each sample, the average dimension of pores was obtained from approximately 120 different measurements. Thermogravimetric analysis (TGA) was carried out by a PerkinElmer TGA 7 instrument at a heating rate of 20 °C/min from 50 to 750 °C under a nitrogen atmosphere. Differential scanning calorimetry (DSC) analysis was performed by a PerkinElmer DSC 8000 at a heating rate of 10 °C/min from 50 to 240 °C under nitrogen flow to assess the completion of the curing reaction in the foams. Mechanical properties of the foams were investigated by compression tests using a ZwickRoell Retro-Line Z010 dynamometer equipped with a load cell of 2.5 kN. Cylindrical-shaped specimens were compressed at a cross-head rate of 5 mm/min until 70% strain at room temperature. Reported data were averaged at least three tests per specimen.

## Results and Discussion

### Microwave Heating Tests

In conventional heating-based material processing techniques, external heating is usually supplied through conduction, convection, and radiation. Temperature gradients are generated during these heating mechanisms, and the different temperatures within the materials may affect chemical and/or physical transformations, thus leading to possible alteration of microstructural and mechanical properties of the final product. Microwave heating of materials, characterized by rapid, volumetric, and uniform heating throughout the thickness of the material, provides several benefits, including lower power consumption and reduced processing time. The use of microwave curing has been explored in the field of epoxy resins and their composite materials since the 90s.^39–46^ However, very scarce literature is available on MW interactions with vegetable oil-based resins, and as far as we know, only one paper has been published on the microwave curing of AESO resins.^26^ For these reasons, we preliminarily investigated the effect of the MW processing conditions on the heating mechanisms of AESO resins with the aim of optimizing the curing conditions during foaming.

Heating mechanisms in microwave processing of materials depend upon the interactions between the electromagnetic characteristics of the MW with the target material. In nonmagnetic materials, such as the case of water, monomers, and polymers, dipolar loss is the principal mechanism for the generation of heat.^45^ Under an oscillating electric field, dipoles realign themselves to be in phase with the field and this rapid dipole orientation generates heat in the material due to the presence of inertial, frictional, elastic, and molecular interaction forces.^47^ During MW heating, the absorption of MW power by a dielectric material can be determined in eq 1:^45^

$$P_{v}=2\pi f\varepsilon_{0}\varepsilon^{’’}E^{2}$$ 1

where *P*_*v*_ denotes the converted thermal energy per unit volume, *f* denotes the MW frequency, ε_0_ denotes the permittivity of free space (constant value, 8.854 × 10^–12^ F/m), ε*″* denotes the dielectric loss factor of the material (relative to free space), and ***E*** denotes the root-mean-square of the electric field.

We performed heating experiments in both a CEM Discover 2.0 single-mode microwave reactor and a commercial household multimode microwave oven. The main differences between the two apparatuses are the following:

- A multimode oven MW field is not constant in the cavity, and there are areas of high and low microwave energy. The sample needs to be rotated to reduce the hot spot. In a single-mode MW reactor, the cavity is designed for the length of only one wave (mode). By placing the sample in the middle of the cavity, it can be irradiated constantly with MW energy.
- Due to the presence of hot and cold spots occurring in a multimode cavity, it is difficult to get constant MW energy to irradiate a small sample. In a monomode apparatus, it is possible to heat very small samples of as little as 0.2 mL efficiently. The upper volume limit of the monomode apparatus is determined by the size of the microwave cavity, which in the CEM apparatus is in a region of 100 mL.
- Multimode microwaves have large cavities, and thus, power is dissipated over a large area. Monomode equipment has a much smaller cavity, and the energy density is up to 30–40 times higher than the multimode apparatus.
- In the monomode apparatus, it is possible to accurately measure the temperature of a reaction (samples do not move inside the cavity) and this can allow for a better control of the power when isothermal or specific rate of temperature changes are necessary.

Figure 1 presents the temperature–time profiles for water, SO, and AESO heated in a monomodal microwave apparatus. These experimental data indicate that both SO and AESO readily absorb microwave energy, exhibiting heating rates comparable to those of water. This observation appears to contradict the significantly lower dielectric loss of vegetable oils compared to that of water, which is attributed to their reduced polarity. Although AESO is expected to possess a higher polarity and thus greater dielectric loss than unmodified soybean oil, its dielectric loss should still be substantially lower than that of water. In a recent work, Zhou et al. have demonstrated, through computer simulation, that vegetable oils can be effectively heated using microwaves, often even faster than water.^48^ Even though vegetable oils have a much smaller ε*″* than water (about 1/100th that of water), the electric field *E* in vegetable oils is much stronger than in water (about 10 times higher), and this compensates for the small loss factor of oil compared to water. Being the converted thermal energy similar in both oil and water, in certain conditions, oil can be heated with an even higher heating rate due to its lower specific heat *c*_p_ (about 1/2 of water).

**Figure 1:**
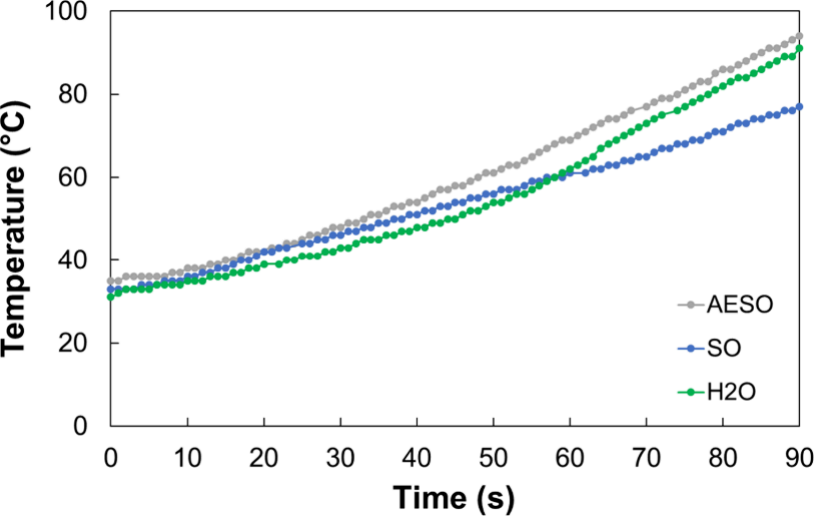
Comparison of the MW heating kinetics for water, SO, and AESO at 50 W. (Tests performed in a MW system CEM Discover 2.0).

Heating experiments for AESO in a multimodal household microwave were conducted by using a cylindrical silicone mold (Figure S1) with a diameter of 4 cm. Sample height was consistently maintained at 0.8 cm, and three distinct microwave power settings were employed: 100, 450, and 800 W. Sample temperature was measured using a thermal camera after the mold was removed from the microwave following a defined heating period. The nonuniformity of the electromagnetic field can lead to hot spot formation.^45^ Nevertheless, despite the temperature profiles in Figure 2 being derived from measurements at the sample’s geometric center, we noted only restricted temperature deviations within the sample. For instance, at a power of 100 W, the central temperature was 129 °C, whereas the sample’s peak temperature measured at 133 °C (Figure 2).

**Figure 2:**
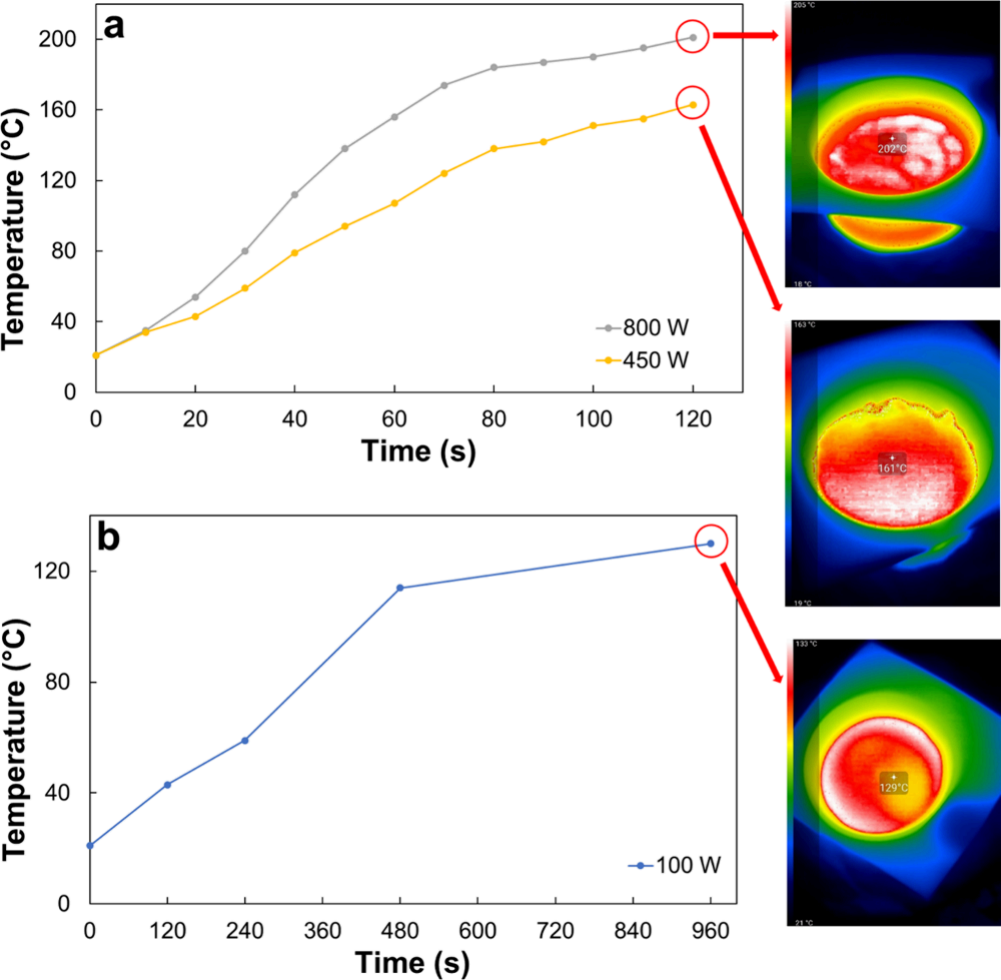
MW heating kinetics for AESO at (a) 450 and 800 W and (b) 100 W.

As shown in Figure 2a, temperature measurements were collected from 0 to 120 s at both 450 and 800 W. At the end of these tests, approximate temperatures of 160 and 200 °C were achieved with 450 and 800 W, respectively. For the lowest power setting (100 W), the temperature was monitored up to 960 s, with data presented in Figure 2b. Unlike the higher power tests, a temperature of only 40 °C was recorded after 120 s. The temperature then nearly stabilized after 480 s, reaching approximately 130 °C after 960 s.

Previous studies on AESO-based thermosetting resins indicate that curing occurs within a temperature window of 100–180 °C.^19,20,24^ Consequently, considering both the observed MW heating kinetics and the established thermal requirements for AESO-based material curing, 800 W was determined to be the optimal MW power for resin curing during the MW-assisted foaming process. As detailed below, these processing conditions facilitate rapid heating of the resin, thereby stabilizing the cellular structure generated by the chemical blowing agent’s decomposition.

### Optimization of Rheological Properties for Precuring

When designing foaming strategies for thermosetting polymers, a critical aspect is the precise control over bubble nucleation and growth as polymerization and cross-linking proceed. The reactive medium’s viscosity, initially low, steadily increases with enhancing molecular weight. Consequently, successful bubble formation and expansion necessitate that nucleation and growth take place within an optimal viscosity range, which prevents bubble collapse. A tight synchronization between cell nucleation and growth and curing kinetics is required. Ideally, after the formation of bubbles, curing should occur as quickly as possible to avoid inevitable collapse or loss of the foaming gas from the resin.

Previous research consistently showed that foam expansion in these systems relies on a precuring strategy where the resin is cured for less than its critical gelation time, thus maintaining a cross-link density low enough for proper expansion.^11,12,22,27,49^ It has been observed that extending the precuring time beyond the critical gelation point generally yields foams characterized by significantly reduced cell sizes and low porosities.

Rheometry is a powerful technique for monitoring changes in the viscoelastic properties of materials during curing.^50^ Specifically, real-time tracking of the storage modulus (*G′*) and loss modulus (*G″*) as a function of temperature or time allows for observation of a sample’s liquid-to-solid transition. The time-dependent evolution of *G′*, *G″*, and complex viscosity (η*) during the curing of AESO was investigated using both nonisothermal (Figure 3) and isothermal experiments (Figure 4).

**Figure 3:**
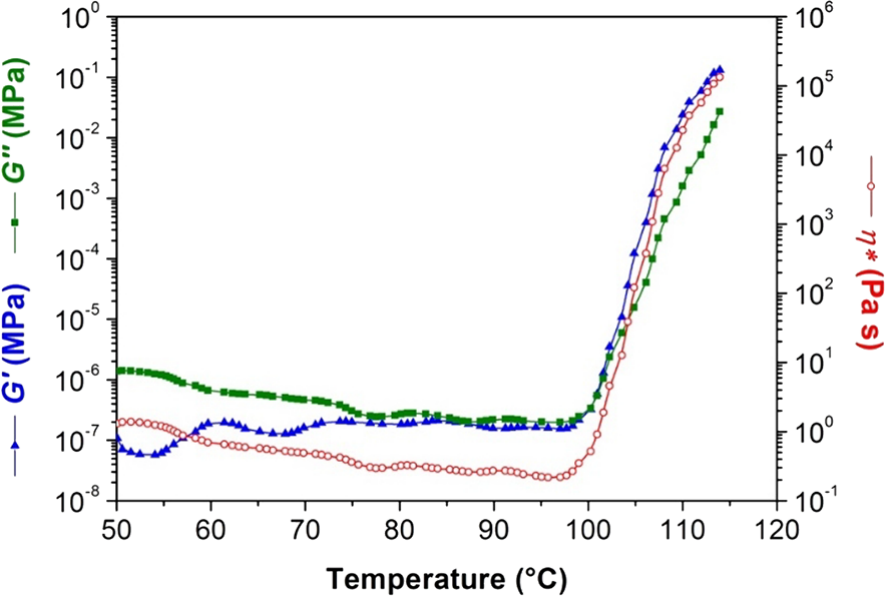
Storage and loss moduli and complex viscosity as a function of temperature for AESO.

**Figure 4:**
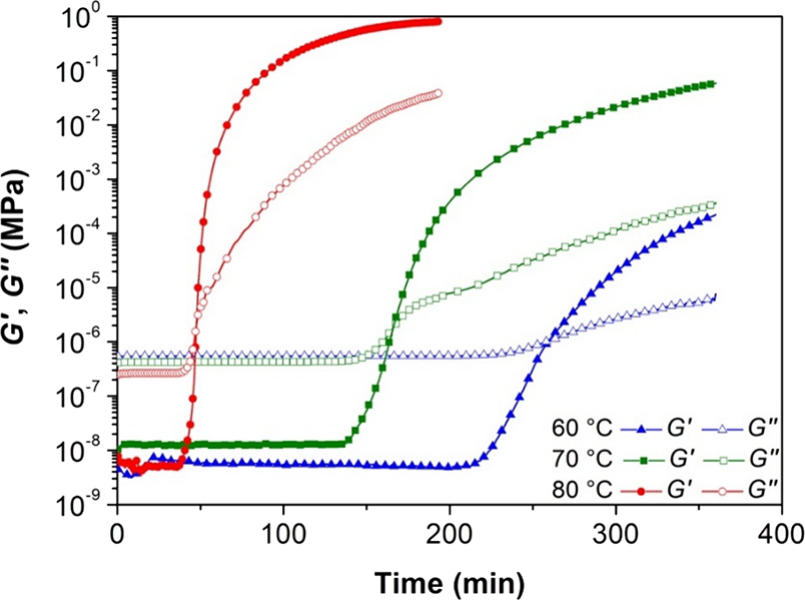
Storage and loss moduli as a function of time at 60, 70, and 80 °C for AESO.

During the nonisothermal experiment (Figure 3), the complex viscosity decreased by approximately 1 order of magnitude as the sample was heated from 50 to 115 °C. Within this temperature range, the curing kinetics are relatively slow, allowing the increase in molecular mobility, driven by rising temperature, to be the dominant factor. Between 100 and 115 °C, a distinct acceleration in curing was observed, manifested by sharp increases in *G′*, *G″*, and η*. Consistent with network formation, *G′* exhibited a more rapid increase than *G″*, indicative of the transition from a viscous fluid to an elastic solid. The gel point is conventionally defined by the crossover of *G′* and *G″* ^50^ and occurs at 100 °C after 12 min of reaching a modulus value of 8 × 10^–7^ MPa.

Based on these findings, isothermal experiments were carried out within the 60–80 °C range to optimize precuring conditions. In this window, the curing kinetics are sufficiently slow (Figure 4 and Figure S2) to allow for better control of AESO precuring. Moreover, these temperatures are well below the decomposition point of the chemical blowing agent intended for MW-assisted resin foaming. As in previous nonisothermal experiments, *G′* and *G″* increased after an initial induction period, indicating progressive chain growth and network formation. In the latter stages, both *G′* and *G″* continued to rise with time, which is attributed to the slower increase in the degree of cross-linking.

Higher temperatures for isothermal curing had three primary effects: (a) a decrease in the induction period; (b) a reduction in the time required to reach the gel point; (c) an increase in the final *G′*, *G″*, and η*.

As detailed in Table 1, all characteristic times, that is *t*_I_ (induction time), *t*_G_ (gelation time), and *t*_G_–*t*_I_, decreased with increasing temperature. This unequivocally indicates an accelerated curing reaction rate with the increase in temperature. Furthermore, an elevated curing temperature led to higher values of *G′* and *G″* at the crossover point. This suggests that the molecular network of the resulting gel is characterized by a greater number of cross-linking points, likely due to the higher concentration of radicals generated by the initiator when increasing temperature. This is consistent with the significantly higher elastic modulus observed at the end of the tests. Given these results, precuring was performed at 80 °C since at this temperature, the curing kinetics is sufficiently slow to allow for a good control of the network close to the gel point of the resin.

**Table 1:** Rheological Data of the Isothermal Curing^a^

temperature (°C)	*t*_I_ (min)	*t*_G_ (min)	*t*_G_–*t*_I_ (min)	*G′* and *G″* at *t*_G_ (10^–6^ MPa)
60	214	259	45	1.03
70	136	166	30	1.79
80	37	48	11	2.05

a *t*_I_ (induction time), *t*_G_ (gelation time), *t*_G_–*t*_I_ (the onset of significant change before gelation) and *G′* and *G″*at the gel point

### Preparation and Characterization of MW-Assisted AESO Foams

Sodium bicarbonate, a well-known nontoxic foaming agent, decomposes endothermically producing carbon dioxide, water, and sodium carbonate (Na_2_CO_3_). TGA analysis of NaHCO_3_ showed an onset decomposition temperature (*T*_d_) of 120 °C, determined in correspondence of a mass loss of 2%, a maximum rate degradation temperature (*T*_max_) of 164 °C, measured by the peak of the derivative thermogravimetric curve (DTG), and a solid fraction of residual material of 62% attributed to Na_2_CO_3_ (Figure S3).

Effective development of a porous foam structure relies on the synchronized decomposition of NaHCO_3_ and the cross-linking of the AESO. A rapid curing kinetics is thus paramount to restrict cell coalescence and prevent the loss of gas from the resin matrix. To address this, a maximum microwave power of 800 W was applied, ensuring a high heating rate. Under these operational parameters, the precured AESO resin attains temperatures of 140 °C after 50 s and 200 °C after 2 min.

To investigate the role of precuring on foaming behavior, three distinct precuring conditions were analyzed: (i) 30 min at 80 °C, a stage where the resin remained within its induction time (sample A1); (ii) 46 min at 80 °C, corresponding to a state below the resin’s gel point (sample A2); and (iii) 60 min at 80 °C, where the resin is well beyond its gel point (sample A3). As presented in Figure 5, the complex viscosity demonstrated an approximate 2 order of magnitude increase over an 8 min interval leading up to its gel point (indicated by the red line). During this specific time frame (between 40 and 48 min), the rate of viscosity evolution proved to be adequately manageable, thereby permitting the successful preparation of precured AESO formulations near their gelation threshold. Precured samples A1, A2, and A3 are indicated by the red circles in Figure 5.

**Figure 5:**
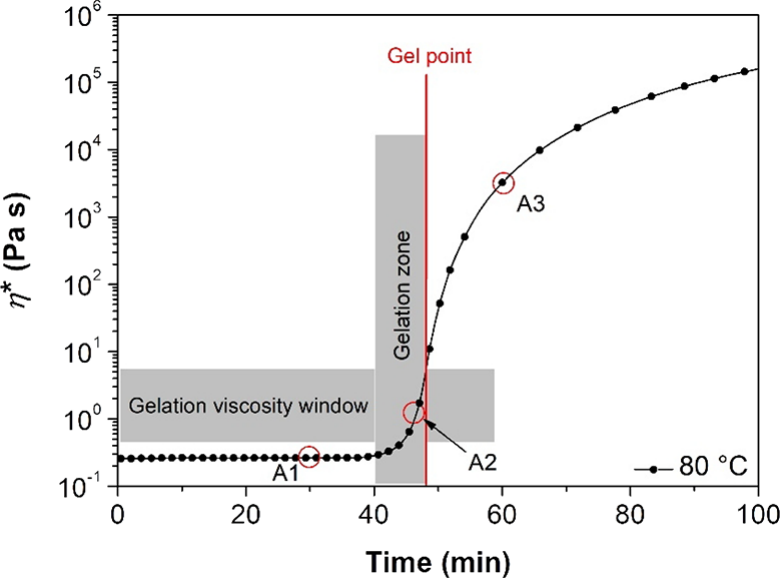
Evolution of complex viscosity (η*) of AESO at 80 °C. Precured samples A1, A2, and A3 are indicated by the red circles.

Figure 6 shows some representative SEM micrographs of samples A1, A2, and A3, taken from the transversal plane to the foam growth. When the extent of precuring is below the induction time, the cellular structure is characterized by a limited number of large-diameter cells (Figure 6a). Under this condition, the viscosity of the resin is low and coalescence of neighboring cells predominates. As the extent of precuring increases but remains below the gelation point, the improved viscoelastic properties facilitate a better stabilization of the cells during their growth. A prevalence of an open-cell structure with an average pore diameter (*d*) of 190 μm was observed (Figure 6b). Conversely, when precuring proceeds significantly beyond the gel point, both the nucleation and growth of gas bubbles are hindered and occur prevalently at the bottom of the foam (Figure 6c).

**Figure 6:**
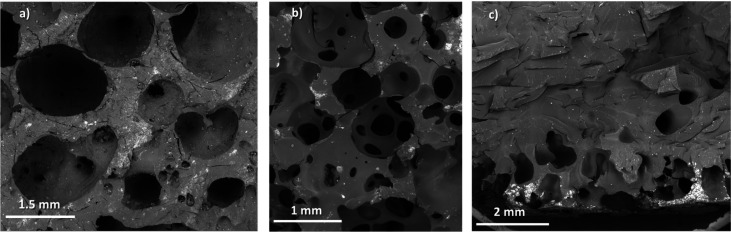
SEM morphologies of AESO foams with different precuring times: (a) 30 min, well below the resin’s gel point, where the resin is in its induction time (A1), (b) 45 min, slightly below the resin’s gel point (A2), and (c) 60 min, above the resin’s gel point (A3).

The porosities (Φ) of samples A1 (Figure S4a,b) and A2 (Figure S4c,d), calculated using eq 2, were, respectively, equal to 17 and 65%. In eq 2, ρ* is the apparent density of foam and ρ_s_ (1.055 g/cm^3^) is the density measured for AESO resin prepared by conventional thermal curing used in our previous work.^24^

$$\Phi =\left[1-\frac{\rho^{*}}{\rho_{s}}\right]$$ 2

AESO foams were further characterized to assess their thermal and mechanical properties. DSC heating scans showed no exothermic peak, suggesting that, under MW heating at 800 W for 2 min, all cross-linking reactions were completed (Figure S5). TGA-DTG thermograms revealed the good thermal stability of both AESO foams, characterized by a primary degradation stage between 300 and 550 °C, which is similar to the thermal behavior observed in AESO foam reported in the literature.^27^ The maximum degradation rate occurred at 433–435 °C (Figure 7). Furthermore, a residual mass of ca. 5% remained at the end of the test, attributed to both the carbonaceous residue and Na_2_CO_3_, formed during the foaming agent’s decomposition.

**Figure 7:**
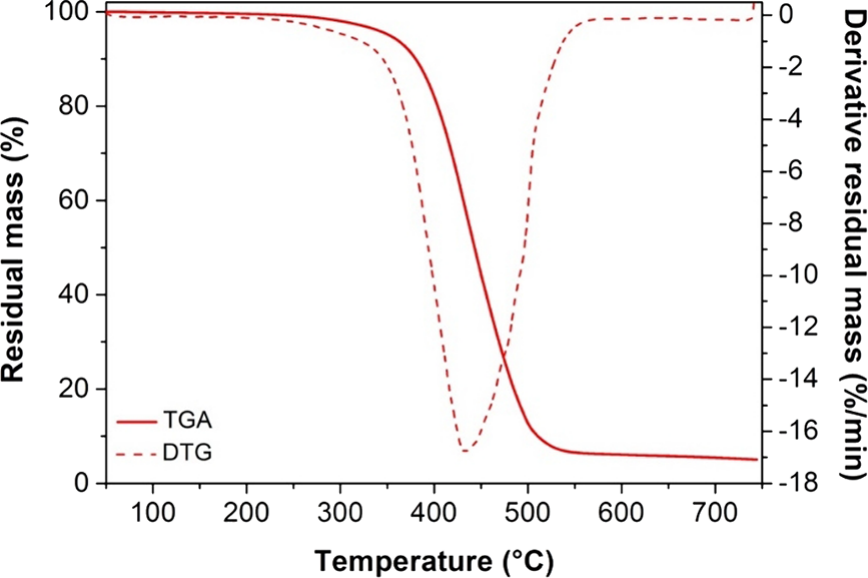
TGA and DTG thermograms of AESO foam A2.

Uniaxial compression tests were performed on the foam along its growth direction to determine its mechanical properties; representative stress–strain curves of foams A1 and A2 are presented in Figure 8. Young’s modulus (*E*_c_) and yield stress (σ_y_), derived from these tests, are detailed in Table 2; the samples present mechanical properties typical of rigid polymeric foams. In particular, the stress–strain curve of foam A1 was characterized by a sharp increase in stress up to 20% strain, which was attributed to the densification of the cellular structure. Testing was concluded at this strain level, as the maximum normal force capacity of the load cell was attained.

**Table 2:** Physical Properties of AESO Foams^a^

sample	ρ*(g/cm^3^)	Φ (%)	*d* (μm)	*T*_d_ (°C)	*T*_max_ (°C)	*R* (%)	*E*_c_ (MPa)	σ_y_ (MPa)
A1	0.897 ± 0.045	17	1191 ± 374	299	435	6	17.32 ± 2.91	
A2	0.368 ± 0.054	65	190 ± 65	299	433	5	1.84 ± 0.21	0.25 ± 0.03

a ρ*: Apparent density of foam. Φ: Foam porosity. *d*: Average pore diameter. *T*_d_: Decomposition temperature at a mass loss of 2%. *T*_max_: Maximum rate degradation temperature. *R*: Residual mass at 750 °C. *E*_c_: Young’s modulus. σ_y_: Yield strength.

**Figure 8:**
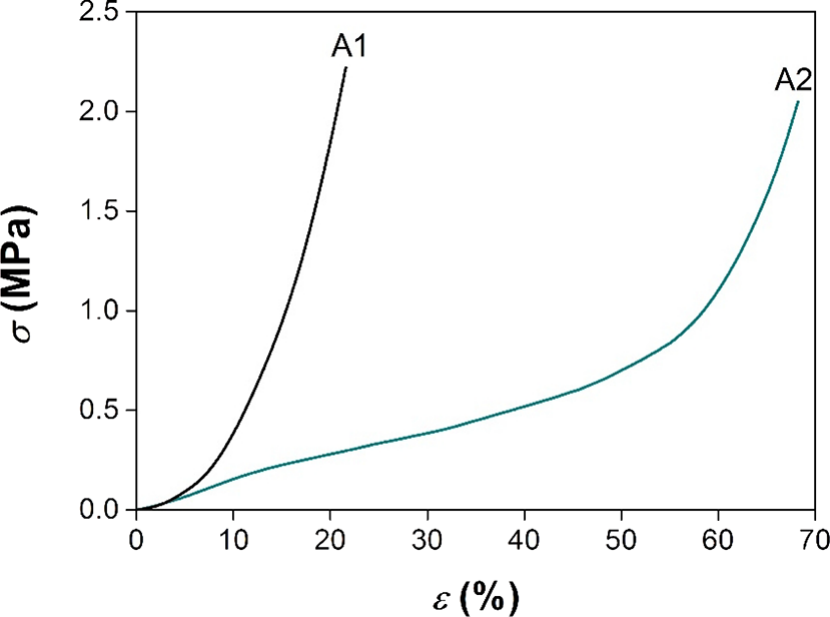
Comparison of compression stress–strain curves of AESO foams A1 (ρ* = 0.897 g/cm^3^) and A2 (ρ* = 0.368 g/cm^3^).

The stress–strain curve of foam A2 instead displayed the characteristic compression behavior of thermosetting foams. Specifically, the AESO foam exhibited linear-elastic behavior at low strains. Upon exceeding the elastic limit, cell collapse occurred, marked by a lower slope of the stress–strain curve. Finally, foam densification was observed above 50% strain, where the stress increased significantly, reaching a value of 2 MPa at the end of the test.

The mechanical behavior of cellular materials is typically described by power-law relationships between their properties and the relative density. The Gibson and Ashby model^1^ is a fundamental framework for this analysis, proposing that the effective Young’s modulus (E*) and yield strength (*σ_y_* *) of a foam are related to the properties of the solid material (*E*_s_ and *σ_ys_*) and the foam’s relative density (ρ*/*ρ_s_*) in eqs 3 and 4:

$$\frac{E^{*}}{E_{s}}=C_{1}{\left(\frac{\rho^{*}}{\rho_{s}}\right)}^{n}$$ 3

$$\frac{{\sigma_{y}}^{*}}{\sigma_{\mathrm{ys}}}=C_{2}{\left(\frac{\rho^{*}}{\rho_{s}}\right)}^{m}$$ 4

For ideal open-cell foams, the exponents *n* and *m* are typically around 2 and 1.5, respectively, as the deformation mechanism is primarily governed by the bending of cell struts. The constants *C*_1_and *C*_2_ are geometrical factors, often approaching 1 for *C*_1_ and ranging from 0.3 to 0.4 for *C*_2_. Given our foam’s predominantly open-cell structure and relative density of approximately 0.37 g/cm^3^, these theoretical relationships provide a valuable basis for understanding the observed mechanical properties.

To enable the prediction of the foam’s mechanical properties, a bulk AESO sample was prepared by curing under the following conditions: 2 h at 140 °C, 2 h at 160 °C, and 4 h at 180 °C. The tensile mechanical properties of this fully cured material were then measured, yielding a Young’s modulus of *E*_s_ = 36 MPa and a tensile strength of *σ_ys_* = 4.3 MPa. By assuming *C*_1_ = 1, *C*_2_ = 0.3, *n* = 2, and *m* = 1.5, the estimated mechanical properties of the foam are E* = 4.42 MPa and σ_y_* = 0.27 MPa. While the estimated yield stress is very close to the measured value (*σ_y_* = 0.25 MPa), the elastic modulus of the foam (*E*_c_ = 1.84 MPa) is lower than the estimated value. The mechanical properties of the foam depend on several factors, including cell geometry, defects, and a nonhomogeneous cellular structure. Given these complexities, the good agreement between experimental data and theoretical predictions provides strong evidence that the microwave cross-linking was successfully completed. This finding was also corroborated by DSC analysis of the foam.

Foam A2 shows mechanical properties similar to the AESO foam from Shukla et al.,^27^ which was prepared by conventional heating and a poly­(methylhydrosiloxane) blowing agent. However, the different apparent densities of our foam and Shukla et al.’s foams, 0.368 and 0.762 g/cm^3^, respectively, required a normalized comparison of the mechanical properties (Figure 9). This analysis reveals that our MW-foamed sample A2 exhibits a significant improvement in both specific Young’s modulus and specific compression strength compared to the conventionally prepared AESO foam.

**Figure 9:**
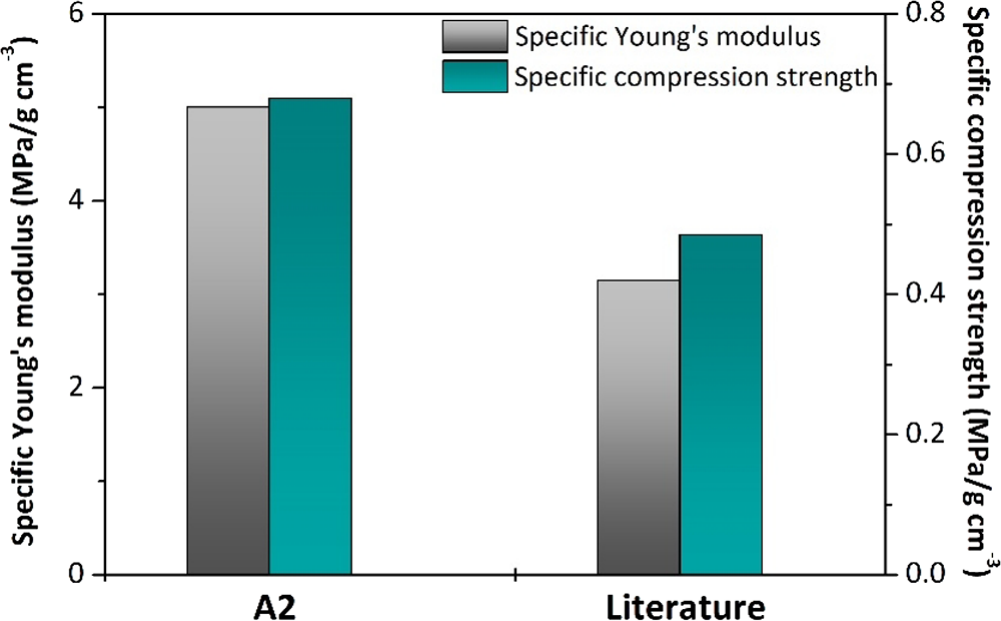
Comparison of specific compression mechanical properties between sample A2 prepared in this work with an apparent density of 0.368 g/cm^3^ and AESO foam with an apparent density of 0.762 g/cm^3^ from literature.^27^

## Conclusions

This study explores the effectiveness of microwaves as a rapid and efficient heating method for foaming acrylated epoxidized soybean oil (AESO) resins. We observed that, despite AESO resin possessing a lower dielectric loss compared to water, its heating rate in a laboratory-scale monomode microwave apparatus is comparable to, and in some cases even higher than, that of water. The use of a common multimode household microwave oven allowed for heating AESO up to 200 °C. This temperature is suitable for simultaneously initiating the decomposition of chemical blowing agents and the cross-linking reaction.

Rheometric analysis conducted under nonisothermal conditions revealed that the cross-linking of the AESO, promoted by a peroxide, proceeds at a high rate in the temperature range of 100–120 °C. During this phase, the complex viscosity of the resin increases by approximately 6 orders of magnitude, indicating rapid solidification of the material. The influence of temperature on cross-linking kinetics was further investigated through isothermal rheometric tests. These experiments demonstrated the feasibility of controlled precuring of the AESO resin at 80 °C, allowing for the achievement of various degrees of polymerization both before and above its gel point.

Foams produced by irradiating the resin with microwaves for 2 min at maximum power (800 W) exhibited significantly different cellular morphologies as a function of the degree of precuring. The most performant cellular structure, characterized by lower density and cell size, was obtained using resin precured just below its gel point. This foam presented an apparent density of 0.368 g/cm^3^, an average cell diameter of 190 μm, and an elastic modulus of 1.84 MPa, parameters that attest the good mechanical and morphological properties of this cellular material.

## Supplementary Material



## Notes

Silicon mold filled with AESO; complex viscosity as a function of time at 60, 70, and 80 °C for AESO; TGA and DTG thermograms of sodium bicarbonate; images of foams A1 and A2; and DSC thermogram of foam A2 (PDF)
