# Supplementary material for: Cellular Structure Optimization in Microwave-Assisted Foaming of Acrylated Epoxidized Soybean Oil
Source: ACS Omega. 2025 Nov 13;10(46):56715–23. doi: 10.1021/acsomega.5c09441 (PMC12658802; doi:10.1021/acsomega.5c09441)
Supplement: Supplementary file 1 [file ao5c09441_si_001.pdf]

Supporting Information

Cellular Structure Optimization in Microwave-assisted Foaming of Acrylated Epoxidized Soybean Oil

Adriano Vignali*, Fabio Bertini and Salvatore Iannace

Institute of Chemical Sciences and Technologies "Giulio Natta" (SCITEC), National Research Council (CNR), Via A. Corti 12, 20133 Milano, Italy

*Corrisponding Author

Email: adriano.vignali@scitec.cnr.it

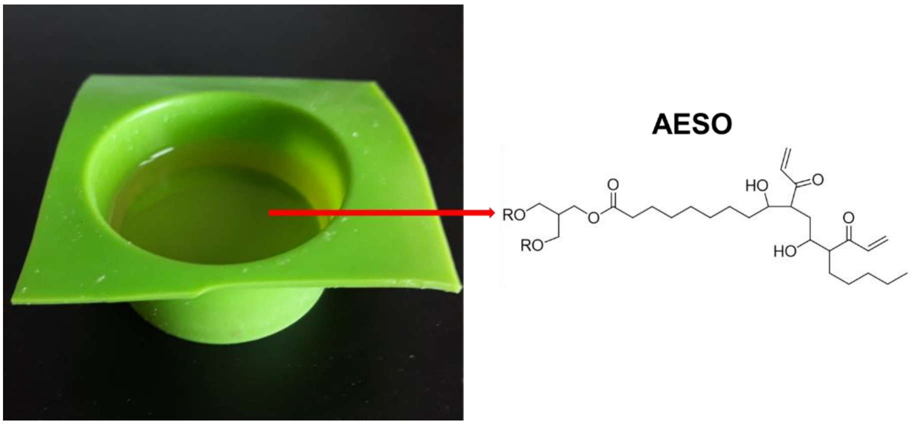

Figure S1. Silicon mold filled with AESO.

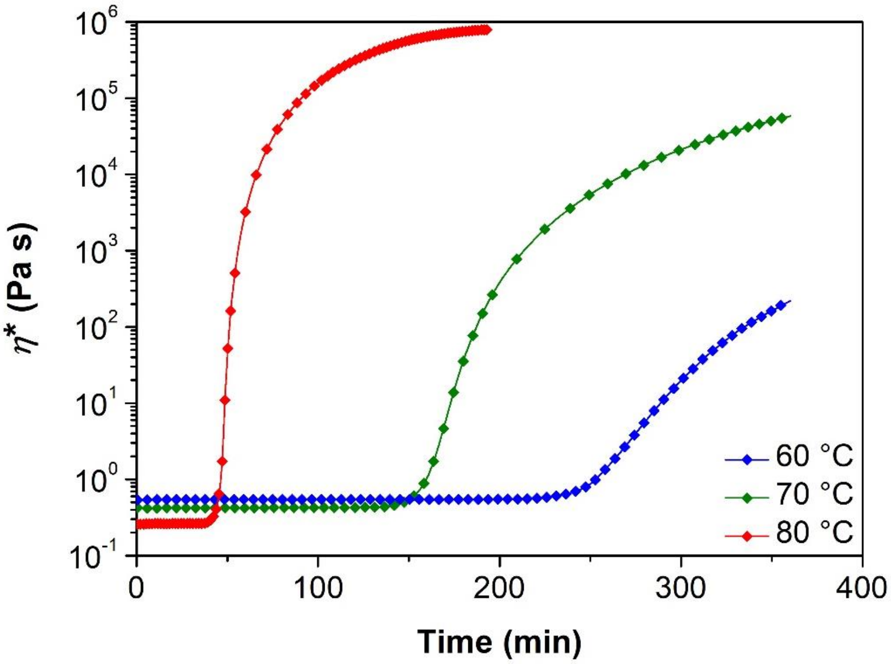

Figure S2. Complex viscosity as function of time at 60, 70 and 80 °C for AESO.

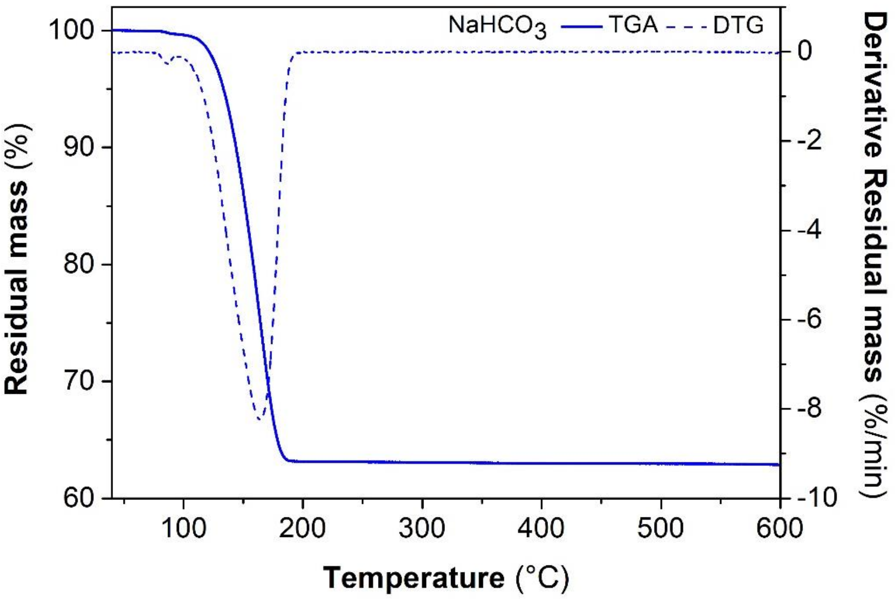

Figure S3. TGA and DTG thermograms of sodium bicarbonate.

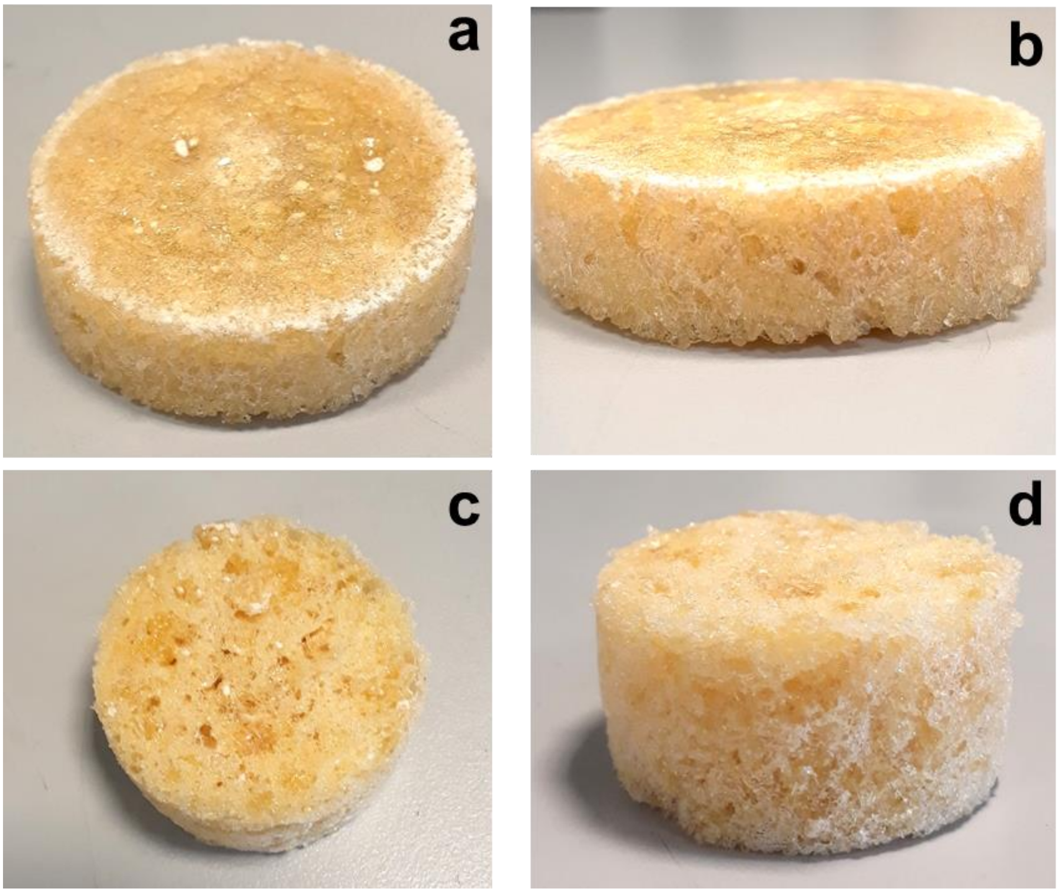

Figure S4. Images of foam: A1 (a,b) and A2 (c,d).

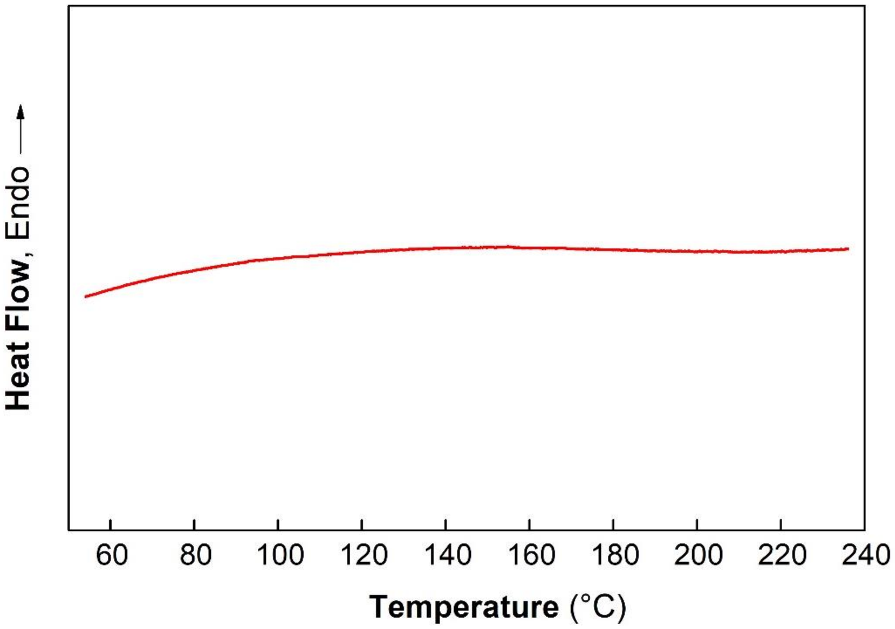

Figure S5. DSC thermogram of foam A2.
